# Short-term modulation of human milk oligosaccharides in plasma and milk by glucose and insulin: insights into postpartum metabolic regulation

**DOI:** 10.1007/s00125-026-06729-y

**Published:** 2026-04-17

**Authors:** Lukas Schönbacher, Anna M. Walzl, Christina Stern, Harald C. Köfeler, Harald Sourij, Herbert Fluhr, Evelyn Jantscher-Krenn, Maria A. Ramos-Roman

**Affiliations:** 1 Department of Obstetrics and Gynecology, Medical University of Graz, Graz, Austria; 2 BioTechMed-Graz, Graz, Austria; 3 Center for Medical Research, Medical University of Graz, Graz, Austria; 4 Department of Internal Medicine/Division of Endocrinology and Diabetology, Medical University of Graz, Graz, Austria; 5 Department of Internal Medicine/Endocrinology, UT Southwestern Medical Center, Dallas, TX USA

**Keywords:** Gestational diabetes mellitus (GDM), Glucose, Human milk oligosaccharides (HMOs), Hyperinsulinaemic–euglycaemic clamp, Insulin, Lactation, Metabolism, Oral glucose tolerance test (OGTT)

## Abstract

**Aims/hypothesis:**

Lactation is associated with reduced maternal risk of future diabetes mellitus and cardiovascular disease. Human milk oligosaccharides (HMOs), bioactive glycans produced in the mammary gland and already detectable in the maternal circulation during pregnancy, are hypothesised to exert endocrine and metabolic effects. We investigated circulating HMOs in the postpartum period, and assessed their short-term modulation by glucose and insulin, and the relationship between plasma and milk HMO profiles.

**Methods:**

At 5–7 weeks postpartum, 28 women (16 with prior gestational diabetes [GDM]; 18 who were breastfeeding) underwent both a 75 g oral glucose tolerance test (OGTT) and a hyperinsulinaemic–euglycaemic clamp. HMOs were quantified in plasma and milk using HPLC.

**Results:**

Seventeen HMOs were detected in milk, of which six were also detected in plasma at concentrations that were approximately 10,000-fold lower but were highly correlated across the two compartments. The lactosamine-based glycans 3′-sialyllactosamine (3′SLN) and 6′-sialyllactosamine (6′SLN) were only found in plasma. Lactation status had no significant impact on plasma HMO levels, except for a lower 6′SLN level in breastfeeding women. Women with prior GDM showed lower HMO concentrations in milk when fasting. Plasma HMOs exhibited short-term changes during both tests: during the OGTT, the levels of the fucosylated HMOs 2′-fucosyllactose (2′FL), lacto-*N*-fucopentaose 1 (LNFP1) and lacto-*N*-difucohexaose (LNDFH) significantly decreased, while that of 3′-sialyllactose (3′SL) increased; during the clamp, the levels of all fucosylated HMOs and 3′SL declined, whereas that of 3′SLN increased with rising insulin levels. In milk, only the levels of 2′FL and lactodifucotetraose (LDFT) decreased significantly during the clamp. During the clamp, the area under the curve (AUC) of plasma oligosaccharides partly correlated with BMI and metabolic clearance rate.

**Conclusions/interpretation:**

Circulating HMOs persist during lactation and are acutely regulated by glucose and insulin. The differential response of fucosylated and sialylated species suggests metabolic regulation of HMO biosynthesis, secretion or clearance. These findings support the concept of HMOs as candidate signal molecules in maternal metabolism, linking lactation with postpartum metabolic adaptation.

**Graphical Abstract:**

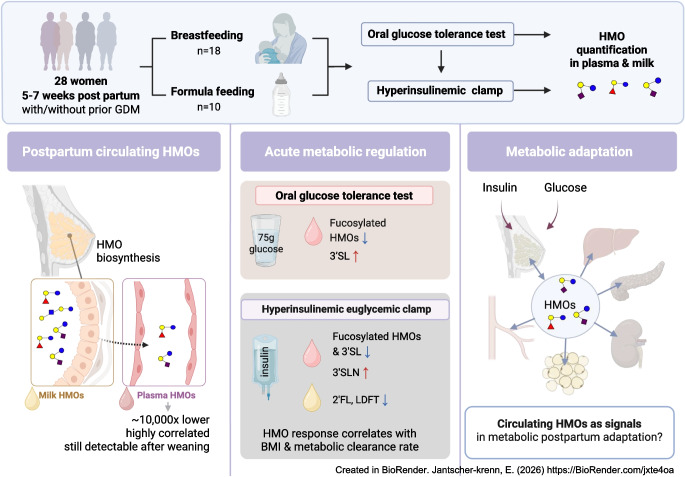

**Supplementary Information:**

The online version contains peer-reviewed but unedited supplementary material available at 10.1007/s00125-026-06729-y.


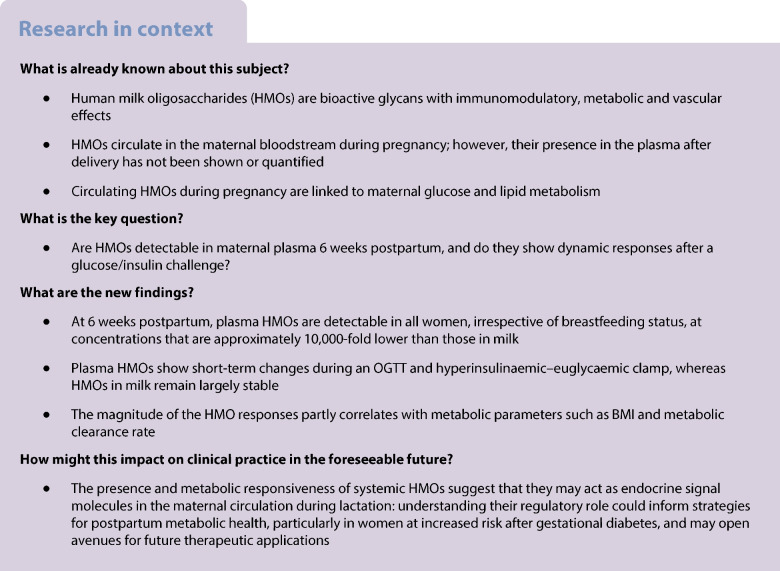


## Introduction

Human milk oligosaccharides (HMOs) are a family of more than 150 distinct bioactive glycan structures. Beyond established prebiotic [1], immune-modulating [2–4] and anti-infective effects [5, 6] in the breastfed infant, emerging evidence suggests roles in maternal metabolic and vascular regulation during the peripartum period.

Lactation imposes substantial metabolic demands and is associated with improved insulin sensitivity and reduced long-term risk of type 2 diabetes and cardiovascular disease [7, 8], which may be particularly relevant for women with hyperglycaemic disorders in pregnancy who remain at increased postpartum cardiometabolic risk [9, 10]. Bioactive milk components, including HMOs, may contribute to this metabolic remodelling. HMOs are also present in the maternal circulation during pregnancy, cross the placental barrier and reach the fetal compartment [11, 12]. Experimental data suggest that they may have immunometabolic and vascular functions, including modulation of inflammatory signalling [4, 13], endothelial cell activation [12, 13] and leukocyte interaction [3]. HMO functions are closely linked to their structural motifs [14]. For example, fucosylated HMOs reduced hepatic de novo lipogenesis and insulin resistance in animal experiments [15], while sialylated HMOs may modulate systemic inflammation by influencing leukocyte–endothelial cell interactions and immune cell activation [16].

HMO biosynthesis in the Golgi apparatus of lactating mammary epithelial cells starts with the formation of lactose [17], which can be elongated by galactose/*N*-acetylglucosamine disaccharide units to form extended backbone structures. Further diversification arises through the action of specific fucosyltransferases and sialyltransferases, which attach fucose and sialic acid (*N*-acetylneuraminic acid) residues, respectively [14]. Certain lactosamine-based oligosaccharides, i.e. 3′-sialyllactosamine (3′SLN) and 6′-sialyllactosamine (6′SLN), which are detectable in blood but not in human milk, differ from classical HMOs by having a lactosamine core rather than a lactose core. This indicates a probable extra-mammary origin, possibly involving the breakdown of glycoconjugates [18, 19].

Maternal HMO profiles exhibit both intra-individual and inter-individual variation [19–23], shaped by genetic factors (notably secretor status) and metabolic status. Secretor status determines the activity of fucosyltransferase-2 (FUT2), which is required for the biosynthesis of α1–2-fucosylated HMOs such as 2′-fucosyllactose (2′FL), lactodifucotetraose (LDFT) and lacto-*N*-fucopentaose 1 (LNFP1). Secretor-negative women almost completely lack these HMOs, whereas secretor-positive women produce them abundantly.

Beyond genetic factors, maternal metabolic factors also influence HMO profiles. Circulating HMOs correlate with maternal glucose and lipid metabolism during pregnancy [18, 24, 25]. In particular, serum 3′-sialyllactose (3′SL) has been associated with higher insulin resistance, and its level increases during an oral glucose tolerance test (OGTT) in pregnancy [18]. Moreover, altered HMO profiles have been observed in women with obesity or gestational diabetes (GDM) [26–28], suggesting a link between HMO biosynthesis and maternal metabolic regulation.

Obesity and GDM disrupt insulin signalling, which may also affect the mammary gland, in which insulin-responsive anabolic pathways [29, 30] may be impaired. While GDM typically resolves postpartum, it remains unclear whether these disruptions persist and influence HMOs in milk or plasma in the early postpartum period. Moreover, although HMOs have been linked with glucose metabolism in pregnancy [18], their acute regulation by glucose and insulin in the early postpartum period has not been examined. Understanding whether HMOs dynamically respond to metabolic cues could provide new insights into their potential role as maternal signal molecules during lactation.

We therefore examined the short-term dynamics of HMOs in maternal plasma (*n*=28) during two standardised metabolic tests, an OGTT and a hyperinsulinaemic–euglycaemic clamp, and in milk (*n*=18) during the clamp only, conducted at 5–7 weeks postpartum. We investigated (1) the relationships between plasma and milk HMOs; (2) acute HMO responses to glucose or insulin; and (3) modulation of HMO dynamics by lactation status, prior GDM, or metabolic parameters (BMI, the Matsuda index and the metabolic clearance rate [MCR]). We hypothesised that HMOs function not only as infant nutrients but also as endocrine signals coordinating postpartum metabolic, vascular and immune adaptation.

## Methods

### Study design

**Fig. 1:**
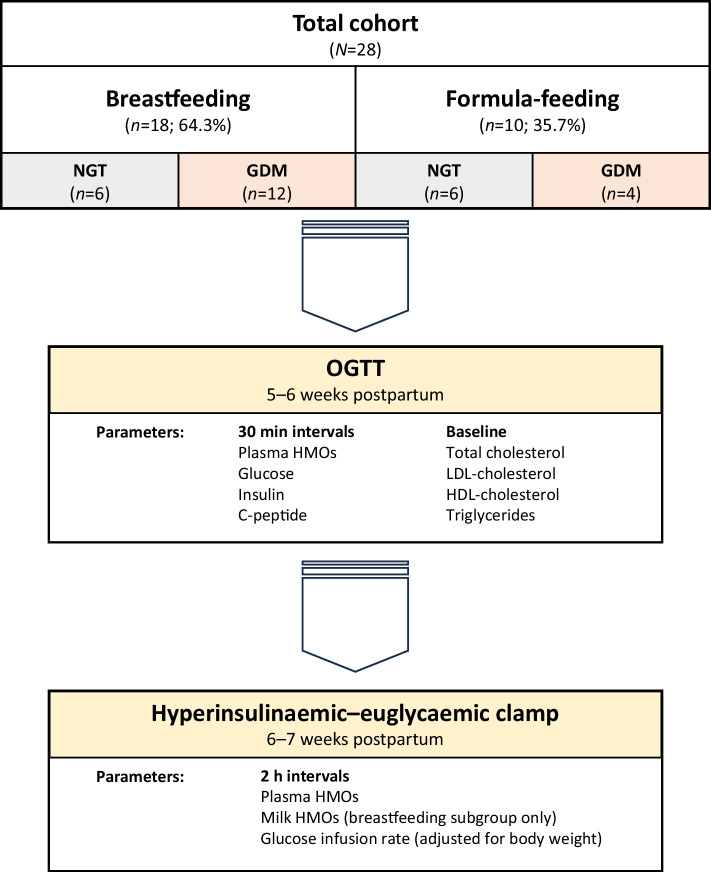
Study flowchart

Figure 1 provides a flowchart of the basic structure of the study, which used samples from a prior study that was primarily designed to investigate the impact of lactation on liver and adipose tissue metabolism [31]. The cohort comprised 28 postpartum women of self-reported Hispanic ethnicity, including breastfeeding (*n*=18, 64.3%) and formula-feeding women (*n*=10, 35.7%) who had prior GDM or normal glucose tolerance (NGT) during pregnancy.

Participants were recruited in the third trimester of pregnancy and/or at a routine visit between 2 and 5 weeks postpartum at Parkland Health & Hospital System, the public health system in Dallas that is affiliated with the University of Texas Southwestern Medical Center, Dallas, TX, USA, and that mostly cares for patients with low income. Over 70% of annual deliveries at Parkland are among Hispanic women. Inclusion criteria were age 21–49 years, singleton term delivery, and BMI 25–35 kg/m^2^ at 2 weeks postpartum. Participants had either NGT or diet-treated GDM that was diagnosed during the third trimester [32]. Based on their self-selected infant-feeding regimen, women were assigned to the breastfeeding group if this was the predominant feeding method, with less than 177 ml of formula (6 fl oz) being used per day. Inclusion in the formula-feeding group required transition to exclusive formula feeding by 3 weeks postpartum. Exclusion criteria included insulin-treated GDM, pre-existing type 2 diabetes, history of pre-eclampsia, use of hormonal contraception, use of medications that interfere with nutrient metabolism, liver or kidney disease, uncontrolled hypothyroidism and postpartum depression. The study was conducted in accordance with the Declaration of Helsinki, and was approved by the University of Texas Southwestern Institutional Review Board (number STU092010-071). All participants provided written informed consent and underwent OGTT and clamp studies on two separate visits within a 2 week window.

### Visit 1: OGTT

At visit 1, anamnestic data (e.g. age, duration of pregnancy, infant birthweight) and biometric data (e.g. body weight, height, BMI) were obtained. A fasting 75 g 2 h OGTT was performed with plasma sampling at five time points (−10, 30, 60, 90 and 120 min) for glucose, insulin and C-peptide. Milk samples were not collected during the OGTT. Fasting plasma glucose levels were defined as normal if they were below 5.6 mmol/l; impaired fasting glucose (IFG) was defined as fasting plasma glucose values from 5.6 to 6.9 mmol/l. NGT was defined as 2 h plasma glucose <7.8 mmol/l; impaired glucose tolerance (IGT) was defined as 2 h plasma glucose values between 7.8 and 11.0 mmol/l. Diabetes mellitus was defined as fasting plasma glucose levels ≥7.0 mmol/l or 2 h plasma glucose values ≥11.1 mmol/l [32]. Additionally, plasma lipids (total cholesterol, HDL-cholesterol, LDL-cholesterol and triglycerides) were measured when fasting using commercial enzymatic kits. Plasma was either analysed promptly (for insulin/glucose/lipid parameters) or stored at −80°C (for HMOs). The Matsuda index [33] was calculated based on the OGTT data.

### Visit 2: hyperinsulinaemic–euglycaemic clamp

At visit 2, participants underwent a hyperinsulinaemic–euglycaemic clamp with stable isotope infusion, as previously described [31]. Fasting participants had two intravenous lines placed (one for infusion of test substances and the other one for blood draws). The 6 h test consisted of three stages of 2 h each. During the basal period (0–120 min), the participant received no exogenous insulin. This was followed by a low-dose insulin infusion (10 mU m^–2^ min^–1^; 120–240 min) and then a medium-dose insulin infusion (20 mU m^–2^ min^–1^, *n*=18; 240–360 min) or a high-dose insulin infusion (40 mU m^–2^ min^–1^, *n*=10; 240–360 min). Plasma glucose was measured every 5 min, and the glucose infusion rate was adjusted to maintain a target clamp glucose level of 4.4 mmol/l.

Milk samples were only collected during the clamp, not during the OGTT. Breastfeeding women pumped milk for 20 min at the beginning of each study phase (0–20, 120–140, 240–260 min) and once at the end of the study for 10 min (360–370 min). These time points represent the fasting condition and the steady state for each step of the protocol. The intention was to standardise the duration of the lactation stimulus at the start of every 2 h step. Milk and plasma samples were stored at −80°C for HMO measurements. The MCR (ml/m^2^/min) was calculated for the clamp as insulin infusion rate [µU/m^2^/min]/(insulin step 1 [µU/ml] − baseline insulin [µU/ml]), as described by DeFronzo et al [34].

### HMO isolation from blood plasma

Oligosaccharides were isolated based on a modified protocol previously reported for serum [24]. Plasma (20 µl) was diluted with water containing an internal standard (linear B6-trisaccharide, Dextra Laboratories), extracted twice with chloroform/methanol (2:1), and labelled with 2-aminobenzamide, as previously described [35].

### HMO isolation from milk

For milk analysis, 5 µl milk was diluted with water containing linear B6-trisaccharide (Dextra Laboratories) as an internal standard. Lipids, proteins and salts were removed using solid phase extraction (C18 and Carbograph, Thermo Fisher Scientific) as previously described [36]. Isolated HMOs were dried in a vacuum concentrator, and labelled with 2-aminobenzamide, as previously described [35].

### HMO analysis by HPLC

Labelled samples were analysed by HPLC using a TSKgel Amide-80 column (Tosoh Bioscience, Japan) and a 50 mmol/l ammonium formate/acetonitrile buffer system (linear gradient). HMO peaks were visualised using a fluorescence detector at 360 nm excitation and 425 nm emission, and identified by comparison with commercially available standards (Prozyme, Hayward, CA, USA) for 19 oligosaccharides, including 17 classical HMOs and two lactosamines (indicated by asterisks). The following list is sorted by HPLC retention times: 2′-fucosyllactose (2′FL), 3-fucosyllactose (3FL), 3′-sialyllactosamine* (3′SLN), lactodifucotetraose (LDFT), 3′-sialyllactose (3′SL), 6′-sialyllactosamine* (6′SLN), 6′-sialyllactose (6′SL), lacto-*N*-tetraose (LNT), lacto-*N*-neo-tetraose (LNnT), 3′-sialyl-3-fucosyllactose (3′S-3FL), lacto-*N*-fucopentaose 1 (LNFP1), lacto-*N*-fucopentaose 2 (LNFP2), lacto-*N*-fucopentaose 3 (LNFP3), lactosialotetraose a (LSTa), lactosialotetraose b (LSTb), lactosialotetraose c (LSTc), lacto-*N*-difucohexaose (LNDFH), lacto-*N*-hexaose (LNH), disialyllacto-*N*-tetraose (DSLNT).

Concentrations of HMOs were determined from the area under the curve (AUC) of the individual peaks, and normalised to the internal standard (linear B6-trisaccharide).

### Statistical analyses

Statistical analyses were performed using SPSS version 29 (IBM SPSS, Chicago, IL, USA) and GraphPad Prism version 9.1.2 (GraphPad Software, La Jolla, CA, USA). Values for normally distributed data are presented as means and SD. Values for skewed data (e.g. HMO concentrations) are presented as medians and IQR. Two-sided Spearman rank correlations were used to assess correlations between fasting HMO measurements of the OGTT and the clamp experiment. Differences between breastfeeding and formula-feeding women were tested using two-sided Mann–Whitney *U* tests (for skewed HMO data) or two-sided independent-sample *t* tests for normally distributed data. Changes in HMOs during the OGTT and the clamp were tested using Friedman ANOVAs. Significant results were further evaluated using post hoc tests (Dunn–Bonferroni) and corrected for multiple testing.

Oligosaccharide responses during the OGTT and the clamp were summarised using the total AUC, calculated by the trapezoidal method, and log_10_-transformed to improve normality and homoscedasticity. Associations between selected metabolic parameters (postpartum BMI, the Matsuda index, MCR) and plasma oligosaccharide responses were explored using multiple linear regression models, adjusted for age and days since delivery. Due to the limited sample size, models were restricted to a maximum of three clinical predictors. For all statistical tests, significance was defined as *p*<0.05.

## Results

### Characteristics of the study population

Twenty-eight participants underwent an OGTT and a hyperglycaemic–euglycaemic clamp at 5–7 weeks postpartum. Of these participants, 18 were exclusively breastfeeding (64.3%) and the remaining ten were formula feeding (35.7%). Sixteen were classified as having GDM (57.1%), and the remaining 12 were classified as having NGT (42.9%) in pregnancy. Table 1 shows the cohort characteristics and baseline metabolic parameters. The mean age was 34.2 years and the mean BMI was 30.1 kg/m^2^. Based on the postpartum OGTT, two of the 16 women with prior GDM (12.5%) exhibited IFG, and 11 (68.8%) exhibited IGT, but no participant met the diagnostic criteria for diabetes mellitus. The HDL-cholesterol levels were significantly higher in the breastfeeding group compared with formula-feeding mothers.

**Table 1:** Characteristics of the total study population, stratified by feeding regimen and GDM/NGT status

	Breastfeeding (BF)				Formula feeding (FF)				*p* value
Variable	All (*n*=18)	GDM (*n*=12)	NGT (*n*=6)	*p* value	All (*n*=10)	GDM (*n*=4)	NGT (*n*=6)	*p* value	
IFG postpartum	1	1	0	0.34	1	1	0	0.39	0.70
IGT postpartum	9	9	0	<0.01**	3	2	1	0.36	0.32
Diabetes mellitus postpartum	0	0	0	–	0	0	0	–	–
Age, years	34.2 ± 4.2	34.2 ± 3.7	34.1 ± 5.6	0.95	34.2 ± 3.7	37.4 ± 3.1	32.0 ± 2.2	0.02*	0.95
Parity	3.1 ± 0.8	3.3 ± 0.9	2.7 ± 0.5	0.09	2.7 ± 1.3	2.3 ± 1.0	3.0 ± 1.5	0.37	0.46
Duration of pregnancy, days	275 ± 6	276 ± 7	274 ± 6	0.66	277 ± 10	275 ± 4	279 ± 12	0.51	0.53
Postpartum OGTT visit, days	36 ± 4	36 ± 4	35 ± 3	0.39	43 ± 3	42 ± 2	43 ± 4	0.52	<0.01**
Postpartum clamp visit, days	44 ± 5	44 ± 5	42 ± 3	0.38	51 ± 5	50 ± 7	51 ± 4	0.78	<0.01**
Infant birthweight, g	3553 ± 358	3557 ± 398	3545 ± 292	0.94	3379 ± 427	3581 ± 308	3244 ± 465	0.21	0.29
BMI at time of study, kg/m^2^	29.8 ± 4.5	31.1 ± 4.8	27.2 ± 2.3	0.03*	30.6 ± 2.0	31.0 ± 0.7	30.4 ± 2.5	0.60	0.49
Fasting glucose OGTT, mmol/l	4.9 ± 0.3	5.0 ± 0.3	4.7 ± 0.3	0.02*	5.0 ± 0.5	5.2 ± 0.7	4.9 ± 0.3	0.46	0.56
Fasting insulin OGTT, pmol/l^a^	29.9 ± 31.3	36.1 ± 36.8	18.1 ± 12.5	0.14	44.4 ± 22.9	58.3 ± 27.1	34.7 ± 16.0	0.19	0.18
Fasting C-peptide OGTT, nmol/l	0.40 ± 0.26	0.43 ± 0.30	0.33 ± 0.03	0.25	0.50 ± 0.10	0.56 ± 0.03	0.46 ± 0.10	0.05	0.09
HOMA-IR	1.0 ± 1.0	1.2 ± 1.2	0.5 ± 0.4	0.12	1.5 ± 0.9	2.0 ± 1.2	1.1 ± 0.5	0.24	0.21
Matsuda index	11.9 ± 9.8	9.7 ± 8.9	16.2 ± 10.8	0.24	6.9 ± 4.4	4.8 ± 3.0	8.3 ± 4.8	0.19	0.08
Total cholesterol, mmol/l	5.12 ± 0.57	5.15 ± 0.67	5.10 ± 0.34	0.85	4.99 ± 1.06	4.81 ± 1.32	5.12 ± 0.98	0.70	0.73
HDL-C, mmol/l	1.32 ± 0.28	1.34 ± 0.31	1.24 ± 0.18	0.41	1.06 ± 0.18	1.16 ± 0.16	1.01 ± 0.18	0.19	0.01*
LDL-C, mmol/l	3.21 ± 0.52	3.23 ± 0.59	3.13 ± 0.28	0.59	3.03 ± 0.80	2.87 ± 0.83	3.13 ± 0.85	0.64	0.53
Triglycerides, mmol/l	1.37 ± 0.60	1.24 ± 0.54	1.62 ± 0.69	0.26	2.02 ± 1.08	1.77 ± 1.07	2.19 ± 1.16	0.63	0.16

Values are *n* for categorical variables (number of women) and mean ± SD for continuous variables

^a^Fasting insulin concentrations for this cohort were reported previously based on the clamp data [60]. The fasting insulin concentrations presented here are based on the OGTT data

The results of independent-sample *t* test are shown for breastfeeding GDM vs breastfeeding NGT, formula-feeding GDM vs formula-feeding NGT, and all breastfeeding vs all formula feeding: *p<0.05, **p<0.01

HDL-C, HDL-cholesterol; LDL-C, LDL-cholesterol

**Fig. 2:**
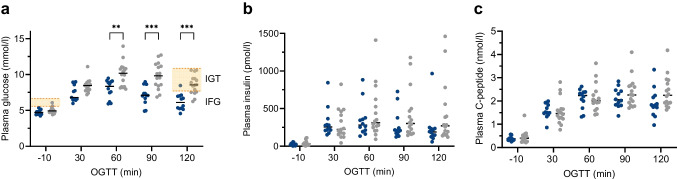
Concentrations of plasma glucose (**a**), plasma insulin (**b**) and plasma C-peptide (**c**) during the OGTT. Interleaved scatter plots show participants with GDM in grey and participants with NGT during pregnancy in blue. The range of IFG (fasting glucose 5.6–6.9 mmol/l at −10 min) and the range of IGT (2 h glucose 7.8–11.0 mmol/l at 120 min) are highlighted in yellow in (**a**). Participants with GDM show significantly higher glucose levels in the OGTT postpartum, with 11 (68.8%) of them fulfilling the criteria for IGT. **p<0.01; ***p<0.001 (*n*=28)

Figure 2 compares plasma glucose, insulin and C-peptide during the OGTT between women with prior NGT and GDM. Fasting glucose did not differ by GDM status; however, glucose at 60, 90 and 120 min was higher in women with prior GDM, indicating delayed clearance. Insulin and C-peptide responses were similar between the groups.

### Fasting plasma HMOs were not different between formula-feeding and breastfeeding mothers

A primary objective of the study was to detect and quantify plasma HMOs postpartum. We were able to quantify six HMOs and two sialyllactosamines (Table 2) in plasma using commercially available standards. Plasma HMOs were detected in all participants, including exclusively formula-feeding women, and tended to be higher in those who were breastfeeding, although the differences were not statistically significant. However, 6′SLN was significantly higher in formula-feeding women (Table 2).

**Table 2:** Concentrations of fasting plasma oligosaccharides during the OGTT according to feeding method

Oligosaccharide	Total (*N*=28)	Breastfeeding (*n*=18)	Formula feeding (*n*=10)	*p* value
2′FL	0.77 (0.44–1.46)	1.13 (0.38–1.70)	0.55 (0.45–0.84)	0.21
3′SLN	0.12 (0.10–0.13)	0.12 (0.10–0.13)	0.12 (0.10–0.14)	0.91
LDFT	0.15 (0.08–0.29)	0.17 (0.09–0.33)	0.12 (0.07–0.26)	0.76
3′SL	0.22 (0.18–0.26)	0.23 (0.20–0.28)	0.20 (0.17–0.24)	0.15
6′SLN	0.08 (0.06–0.13)	0.07 (0.05–0.11)	0.17 (0.08–0.35)	0.006**
LNFP1	0.13 (0.07–0.18)	0.12 (0.06–0.18)	0.14 (0.06–0.19)	0.62
LNFP2/3	0.03 (0.00–0.07)	0.05 (0.00–0.12)	0.02 (0.00–0.04)	0.12
LNDFH	0.05 (0.02–0.11)	0.05 (0.02–0.12)	0.03 (0.02–0.11)	0.55

Values are medians (IQR) (nmol/ml)

The subgroups were compared using the Mann–Whitney *U* test: **p<0.01

### HMOs in milk are reduced in women with prior GDM and are highly correlated with plasma HMOs

To address potential effects of GDM, we assessed fasting milk HMO levels in the GDM/NGT subgroups. Milk samples were only collected at study visit 2 (clamp), not during the OGTT. Table 3 shows the baseline concentrations of 17 HMOs detected in milk; the lactosamines were not found in milk. Three of the 18 women lacked α1–2-fucosylated HMOs (2′FL, LDFT and LNFP1) and were considered secretor-negative; the same women lacked these HMOs in plasma. Median milk HMO concentrations were consistently higher in women without GDM; these differences reached significance for LNnT, LSTb, LSTc, LNH1 and DSLNT.

**Table 3:** Concentrations of baseline (fasting) milk HMOs according to glucose tolerance status during pregnancy

Oligosaccharide	Total (*N*=18)	GDM (*n*=12)	NGT (*n*=6)	*p* value
2′FL	9.50 (5.60–13.34)	8.93 (5.80–12.89)	11.76 (3.94–13.35)	0.82
3FL	0.67 (0.35–0.87)	0.65 (0.32–0.93)	0.70 (0.57–1.35)	0.68
LDFT	0.78 (0.28–1.09)	0.64 (0.28–0.79)	1.07 (0.67–1.28)	0.10
3′SL	0.33 (0.20–0.39)	0.28 (0.19–0.38)	0.36 (0.34–0.46)	0.10
6′SL	0.79 (0.68–1.16)	0.77 (0.52–1.03)	0.97 (0.74–1.27)	0.34
LNT	1.37 (1.09–2.18)	1.25 (0.91–1.57)	2.08 (1.26–2.75)	0.10
LNnT	0.47 (0.34–0.73)	0.44 (0.31–0.57)	0.81 (0.37–1.02)	0.05*
3′S-3FL	0.05 (0.04–0.06)	0.05 (0.04–0.06)	0.06 (0.04–0.10)	0.18
LNFP1	2.20 (0.82–3.08)	1.78 (0.70–2.63)	2.75 (1.49–3.99)	0.29
LNFP2/3	1.11 (0.72–1.43)	1.06 (0.71–1.54)	1.29 (0.87–1.99)	0.55
LSTa	0.21 (0.16–0.24)	0.18 (0.15–0.24)	0.23 (0.21–0.24)	0.21
LSTb	0.20 (0.13–0.28)	0.16 (0.11–0.25)	0.27 (0.20–0.40)	0.03*
LSTc	0.28 (0.21–0.49)	0.25 (0.17–0.33)	0.50 (0.37–0.60)	0.01*
LNDFH	1.11 (0.00–2.25)	0.76 (0.00–1.98)	2.04 (1.27–2.34)	0.18
LNH1	0.02 (0.01–0.03)	0.01 (0.01–0.02)	0.03 (0.02–0.09)	0.02*
LNH2	0.22 (0.18–0.35)	0.21 (0.17–0.36)	0.23 (0.19–0.35)	0.68
DSLNT	0.14 (0.09–0.22)	0.09 (0.08–0.20)	0.18 (0.14–0.37)	0.03*

Values are medians (IQR) (µmol/ml)

The subgroups were compared using the Mann–Whitney *U* test: *p<0.05

**Fig. 3:**
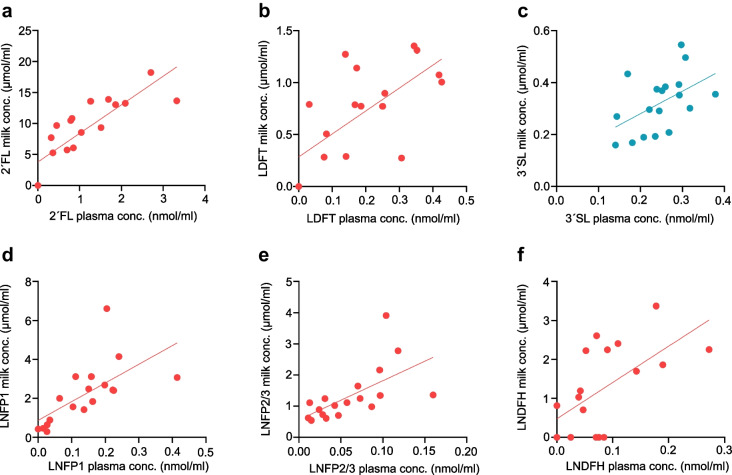
Scatter plots of fasting concentrations (conc.) of HMOs in milk and plasma for the 18 breastfeeding participants. Fucosylated HMOs are indicated in red; sialylated HMOs are indicated in blue. All plots show a highly significant positive Spearman correlation. (**a**) 2′FL (*r*=0.88, *p*<0.001), (**b**) LDFT (*r*=0.66, *p*=0.003), (**c**) 3′SL (*r*=0.55, *p*=0.019), (**d**) LNFP1 (*r*=0.79, *p*<0.001), (**e**) LNFP2/3 (*r*=0.78, *p*<0.001) and (**f**) LNDFH (*r*=0.62, *p*=0.007)

Six HMOs were quantified in both milk and plasma, and showed strong correlations, most prominently for 2′FL (*r*=0.88; *p*<0.001) and LNFP1 (*r*=0.79; *p*<0.001) (Fig. 3a–f). Circulating plasma HMO levels did not differ by GDM status (ESM Table 1).

### HMO dynamics during the OGTT

**Fig. 4:**
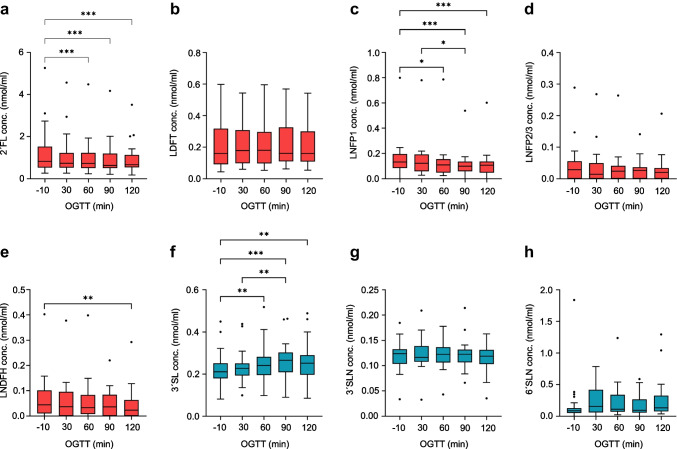
Concentrations (conc.) of plasma HMOs during the OGTT in women at 5–7 weeks postpartum. The box and whisker plots show median, IQR, minimum and maximum values (with the outliers shown separately) for fucosylated HMOs (**a**–**e**) in red, and sialylated HMOs (**f**–**h**) in blue. 2′FL (**a**), LNFP1 (**c**) and LNDFH (**e**) showed a significant decrease after the oral glucose load; 3′SL (**f**) showed a significant increase. The others remained unchanged. Secretor-negative women were excluded from the analysis. *p<0.05; **p<0.01; ***p<0.001 (*n*=25)

As the levels of fasting HMOs in the circulation were not significantly different by breastfeeding status, we used the entire cohort to analyse the potential response of plasma HMOs to a glucose load during an OGTT. Of the eight oligosaccharides measured in plasma, the levels of four changed significantly over the course of the OGTT (Fig. 4a–h). Secretor-negative women (three of the 28 women) were excluded, as α1–2-fucosylated HMOs (2′FL, LDFT and LNFP1) were not detected in their plasma. The fucosylated HMOs 2′FL, LNFP1 and LNDFH significantly decreased from baseline (−10 min) to 120 min, while LDFT and LNFP2/3 showed no significant change. The level of the sialylated HMO 3′SL increased slightly but significantly after the glucose load. The levels of 3′SLN and 6′SLN showed no significant alterations.

### HMO dynamics during the hyperinsulinaemic–euglycaemic clamp

**Fig. 5:**
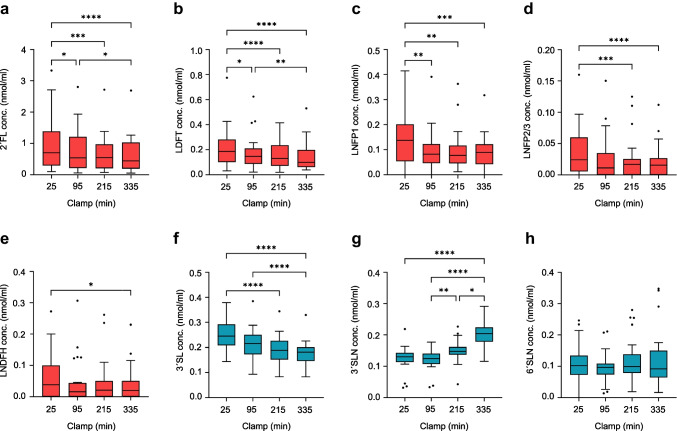
Concentrations (conc.) of plasma HMOs during the hyperinsulinaemic–euglycaemic clamp in women at 5–7 weeks postpartum. The box and whisker plots show median, IQR, minimum and maximum values (with the outliers shown separately) for fucosylated HMOs (**a**–**e**) in red and sialylated HMOs (**f**–**h**) in blue. 2′FL (**a**), LDFT (**b**), LNFP1 (**c**), LNFP2/3 (**d**) and LNDFH (**e**) showed a significant decrease with rising levels of insulin. 3′SL (**f**) also decreases, whereas the corresponding lactosamine 3′SLN (**g**) shows a significant increase. Levels of 6′SLN remained unchanged. Secretor-negative women were excluded from the analysis. *p<0.05; **p<0.01; ***p<0.001 (*n*=25)

We next assessed clamp-induced changes in plasma HMOs (Fig. 5a–h). Again, only secretor-positive women were included. All fucosylated HMOs (2′FL, LDFT, LNFP1, LNFP2/3 and LNDFH) showed significant decreases with increased insulin infusion. In contrast to the OGTT, the level of the sialylated HMO 3′SL also decreased during the clamp. The level of the corresponding lactosamine, 3′SLN, significantly increased, whereas that of 6′SLN remained unchanged. Stratified analyses by feeding group revealed the same trends, but with fewer significant results due to the smaller subgroup size (data not shown).

**Fig. 6:**
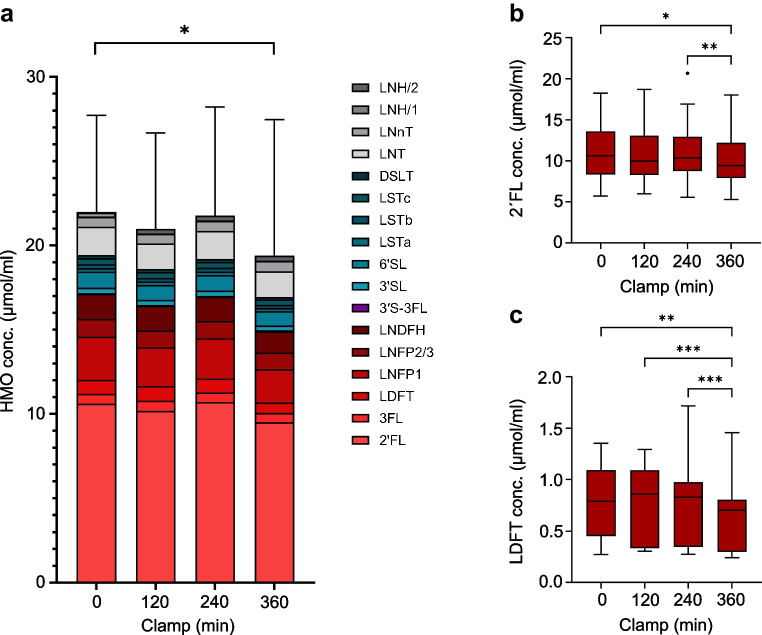
Mean cumulative concentrations (conc.) of HMOs in milk (**a**) and separate concentrations of 2′FL (**b**) and LDFT (**c**) during the hyperinsulinaemic–euglycaemic clamp. Fucosylated HMOs are shown in red colour, sialylated HMOs are shown in blue, and neutral HMOs are shown in grey. The total mean HMO concentration in milk decreases over the course of the experiment, which is mainly due to a reduction of fucosylated HMOs. The box and whisker plots in (**b**) and (**c**) show median, IQR, minimum and maximum values (with the outliers shown separately) for 2´FL and LDFT, revealing a significant decrease of both from fasting status to the last step of the clamp (*p<0.05; **p<0.01; ***p<0.001)

In milk samples collected during the clamp, changes in HMO concentrations were much less pronounced. Seventeen distinct HMOs were quantified, including six of the HMOs also found in plasma, whereas 3′SLN and 6′SLN, which are lactosamine-based and thus are not typical HMO structures, were absent. Consistent with the overall higher concentrations in milk, the HMO profiles showed a broader variety of HMOs compared with plasma, including a wider range of sialylated and neutral HMOs. As shown in Fig. 6a, cumulative mean HMO concentrations in milk significantly decreased from fasting to the high insulin infusion step (*p*=0.02), primarily driven by a significant reduction of fucosylated HMOs (*p*=0.03). Analysis of the individual HMOs in milk revealed no significant changes except for the two highly abundant α1–2-fucosylated HMOs 2′FL and LDFT, whose levels decreased significantly from fasting status to the high insulin infusion step (Fig. 6b, c).

### Associations of changes in plasma HMOs and maternal metabolic traits

We next performed an exploratory analysis to assess whether plasma HMO responses during the OGTT and clamp were associated with metabolic parameters including BMI, the Matsuda index and the MCR. Oligosaccharide responses were summarised as AUCs. In multiple linear regression models (adjusted for age and days since delivery), neither BMI nor the Matsuda index was associated with HMO AUCs during the OGTT (Table 4). During the clamp, the AUCs of LDFT and LNFP1 were associated with BMI, and AUCs of 3′SL and 6′SLN were associated with the MCR (Table 5).

**Table 4:** Multiple linear regression models for the association of plasma oligosaccharide AUCs with BMI and the Matsuda index during the OGTT

	BMI		Matsuda index	
Oligosaccharide	Beta	*p* value	Beta	*p* value
2′FL	−0.29	0.22	0.31	0.15
3′SLN	−0.43	0.08	−0.05	0.84
LDFT	−0.35	0.14	−0.13	0.57
3′SL	0.34	0.15	0.27	0.22
6′SLN	−0.13	0.46	0.10	0.56
LNFP1	−0.30	0.18	0.20	0.34
LNFP2/3	0.28	0.26	0.18	0.42
LNDFH	−0.08	0.76	0.19	0.40

Models are adjusted for age and days since delivery

**Table 5:** Multiple linear regression models for the association of plasma oligosaccharide AUCs with BMI and MCR during the clamp

	BMI		MCR	
Oligosaccharide	Beta	*p* value	Beta	*p* value
2′FL	−0.27	0.22	0.16	0.45
3′SLN	−0.30	0.17	0.11	0.60
LDFT	−0.46	0.03*	−0.20	0.35
3′SL	−0.07	0.76	−0.48	0.02*
6′SLN	0.05	0.84	−0.55	0.01*
LNFP1	−0.56	0.01*	−0.09	0.67
LNFP2/3	−0.13	0.60	0.06	0.78
LNDFH	−0.39	0.07	−0.32	0.13

Models are adjusted for age and days since delivery

^*^p<0.05

## Discussion

In this study, we investigated the concentrations and short-term dynamics of HMOs in the plasma and milk of lactating women and in the plasma of non-lactating women using an OGTT and a hyperinsulinaemic–euglycaemic clamp at 5–7 weeks postpartum. Our key findings are: (1) that HMOs are detectable in maternal plasma irrespective of lactation status, and plasma concentrations correlate strongly with milk levels; (2) that circulating HMOs respond acutely to an oral glucose load, with more pronounced changes during hyperinsulinaemic–euglycaemic clamp conditions, although only 2′FL and LDFT changed significantly in milk; and (3) that BMI and the MCR are associated with some clamp-derived HMO responses.

### Plasma–milk relationship and lactation physiology

Our findings extend previous reports regarding HMOs in the maternal circulation during pregnancy [12, 19, 25] by demonstrating detectable plasma HMOs at 5–7 weeks postpartum even after breastfeeding cessation. Plasma and milk HMO concentrations were strongly correlated, supporting the mammary gland as primary source of circulating HMOs.

Lactation establishment involves the closure of tight junctions in mammary epithelial cells, reducing paracellular permeability [37, 38]. Circulating HMOs during pregnancy and early postpartum are sometimes thought to reflect leakage through these junctions [39]. However, in contrast with expectations of declining plasma HMOs with junction closure, plasma levels were comparable to those in late pregnancy [19]. Women who had discontinued breastfeeding showed slightly lower plasma HMOs compared with breastfeeding women, but the differences were not significant. These findings suggest that, after cessation of breastfeeding, the involuting mammary gland may continue HMO synthesis, and their transfer may be facilitated again by re-opened junctions [37]. As, due to the study design, all participants were living with overweight or obesity, obesity-related alterations in mammary morphology [40] may also contribute.

Although the absolute HMO concentrations were approximately four orders of magnitude lower in plasma than in milk, the relative abundances were consistent, with significant correlations across all six HMOs quantified in both compartments, supporting coordinated regulation between them.

### Acute modulation of circulating HMOs by glucose and insulin

Our data demonstrate that plasma HMO levels are dynamically modulated by glucose and insulin. During the postpartum OGTT, plasma 3′SL increased, consistent with findings in pregnancy [18], while 2′FL, LNFP1 and LNDFH declined, suggesting differential regulation of sialylated vs fucosylated HMOs. In contrast to observations during pregnancy, in which fucosylated HMOs remained stable or increased [18], our findings suggest a different HMO responsiveness postpartum. This difference potentially reflects pregnancy-to-postpartum adaptations in mammary gland function, and associated hormonal and metabolic regulation, as well as study-specific differences. The increased glucose availability during the OGTT may promote sialylation through rapid activation of the hexosamine biosynthetic pathway, which supplies precursors for sialic acid synthesis. During the clamp, the levels of most plasma HMOs, particularly fucosylated species, declined, whereas that of 3′SLN increased. This may reflect its distinct origin, as 3′SLN contains lactosamine rather than lactose and may derive from turnover of glycan conjugates rather than mammary synthesis [18].

Our findings may reflect (1) altered mammary biosynthesis/secretion; (2) as yet uncharacterised insulin-dependent redistribution across tissues; or (3) enhanced systemic clearance (e.g. hepatic or renal). We next speculate on potential mechanisms, although targeted mechanistic studies will be required.

Regulation of HMO biosynthesis remains incompletely understood. After lactose synthesis in the Golgi apparatus of mammary epithelial cells, glucosyltransferases elongate lactose to generate the spectrum of HMOs [39, 41, 42]. Flux through these biosynthetic pathways probably depends on the availability and activity of multiple enzyme systems, precursor metabolites and transporter proteins [43, 44]. Key enzymatic components include UDP-galactose production [44, 45], lactose synthesis [44], the hexosamine pathway [18, 43], ectonucleoside triphosphate diphosphohydrolase 5 (ENTPD5) [46] and multiple glucosyltransferases [47]. Essential precursors comprise monosaccharides, nucleosides, nucleotide sugars, amino acids and metal cofactors, such as manganese [41], requiring dedicated transporters such as GLUT, SGLT, nucleoside transporters, the SLC35 family and metal transporters [46, 48]. Our literature review identified ENTPD5 as a insulin-responsive, hydrolysing UDP/GDP in the Golgi apparatus and ER [49], promoting regeneration of nucleotide sugars required for glycan synthesis [46]. Prolonged insulin during the clamp may therefore favour production of conjugated glycans over unconjugated HMOs.

Insulin may downregulate divalent metal transporter 1 (DMT1), a manganese transporter [50, 51]. As manganese is required for galactosyltransferase activity in lactose synthesis [41], reduced manganese could impair production of lactose and downstream HMOs. However, lactose remained unaltered during hyperinsulinaemic–euglycaemic or hyperglycaemic clamp conditions in women with NGT [52], suggesting that lactose synthesis is robust, although downstream HMO synthesis was not assessed. In our cohort, lower fasting levels of milk HMOs and a decline in milk volume (ESM Table 2) during the clamp in women with prior GDM suggest an influence of alterations in glucose metabolism on lactational glycan regulation.

Very little is known about the systemic redistribution or clearance of HMOs. Renal elimination is plausible, and could be addressed in future studies through concurrent urine analyses. More broadly, dedicated mechanistic investigations will be required to elucidate the regulation, tissue distribution and physiological roles of circulating HMOs across various organs and metabolic states.

### HMO trajectories in milk and association with GDM

With respect to milk HMOs, our analyses did not include milk from the OGTT but were limited to the clamp, which showed a reduction of 2′FL and LDFT towards the end of the experiment when the insulin infusion rates were highest. Measurable changes in milk lag behind acute biosynthetic changes in the mammary epithelial cell, and are therefore probably easier to detect during the 6 h clamp than during a 2 h OGTT. It remains unclear whether blood glucose spikes, such as those that occur during an OGTT, also affect HMOs in milk, but a previous study observed no changes in milk HMOs after a standard meal [53].

As a secondary observation, women with prior GDM showed lower milk HMOs (LNnT, LSTb, LSTc, LNH1 and DSLNT). Although the study was not powered for assessing GDM effects, these findings partly align with previous reports [28, 54, 55]. In women with prior GDM, Li et al observed lower HMOs in colostrum compared to women without GDM, but the differences disappeared in mature milk by day 42 [28]. In our cohort, residual confounding (particularly by the higher BMI in the GDM group) cannot be excluded. However, given the reduction in milk and plasma HMO levels with rising insulin levels during the clamp, the lower HMO levels in women with IGT may reflect effects of chronic hyperinsulinaemia.

### Potential metablic roles of circulating HMOs postpartum and during lactation

Whether systemic HMOs influence maternal metabolism beyond their well-established effects in infants remains unclear. As the metabolic demands of lactation promote long-term maternal metabolic health, including improved insulin sensitivity and reduced cardiovascular risk [8, 10, 56–58], the role of HMOs in this context needs further investigation. Animal studies have demonstrated metabolic effects of orally supplemented HMOs, including 2′FL, on insulin sensitivity and hepatic lipid metabolism [15, 59]. 2′FL reduced hepatic steatosis and fasting insulin concentrations in obese mice [15], although the relative contributions of gut microbiota modulation vs direct systemic effects remain unclear. 3′SL was shown to have anti-atherosclerotic effects in a mouse model [13]. The presence of HMOs in the maternal circulation provides the opportunity to explore their role as endogenous metabolic regulators in vivo. Our findings indicate the need for larger, mechanistic studies to assess whether specific HMOs contribute to postpartum metabolic adaptation or serve as biomarkers of maternal metabolic health.

### Strengths and limitations

This study comprehensively assessed short-term HMO dynamics in plasma and milk using complementary metabolic challenges within the same cohort, including breastfeeding/non-breastfeeding women and those with/without prior GDM. This design allowed the assessment of plasma HMO responses under physiological and tightly controlled metabolic conditions. Limitations include the modest sample size (*n*=28), which especially limited the power of subgroup analyses; thus these results should be interpreted as pilot exploratory results. An a priori power analysis was not feasible due to the absence of comparable studies and thus unavailable information on expected effect size. All participants were living with overweight or obesity, limiting generalisability, but providing insight into a vulnerable group. Milk samples were only available during the clamp, precluding OGTT-related milk analyses.

### Conclusion

Lactation is a unique metabolic state requiring the precise coordination of maternal glucose and insulin metabolism. Circulating HMOs respond dynamically to acute metabolic perturbations, whereas milk HMOs are relatively stable. These findings support modulation of HMOs by maternal metabolic signals, and a potential role in postpartum adaptation. Further studies are required to clarify the regulatory mechanisms and metabolic functions of systemic HMOs.

## Supplementary Information

Below is the link to the electronic supplementary material.

ESM (PDF 296 KB)

## Data Availability

The data that support the findings of this study are not openly available for reasons of sensitivity but are available from the corresponding author upon reasonable request.

## Abbreviations

DSLNT: Disialyllacto-*N*-tetraose
ENTPD5: Ectonucleoside triphosphate diphosphohydrolase 5
2′FL: 2′-Fucosyllactose
3FL: 3-Fucosyllactose
GDM: Gestational diabetes mellitus
GLUT: Glucose transporter
IFG: Impaired fasting glucose
IGT: Impaired glucose tolerance
LDFT: Lactodifucotetraose
LNDFH: Lacto-*N*-difucohexaose
LNFP1: Lacto-*N-*fucopentaose 1
LNFP2: Lacto-*N*-fucopentaose 2
LNFP3: Lacto-*N*-fucopentaose 3
LNH: Lacto-*N*-hexaose
LNnT: Lacto-*N*-neo-tetraose
LNT: Lacto-*N*-tetraose
LSTa: Lactosialotetraose a
LSTb: Lactosialotetraose b
LSTc: Lactosialotetraose c
MCR: Metabolic clearance rate
OGTT: Oral glucose tolerance test
3′S-3FL: 3′-Sialyl-3-fucosyllactose
3′SL: 3′-Sialyllactose
3′SLN: 3′-Sialyllactosamine
6′SL: 6′-Sialyllactose
6′SLN: 6′-Sialyllactosamine
