# Supplementary material for: Short-term modulation of human milk oligosaccharides in plasma and milk by glucose and insulin: insights into postpartum metabolic regulation
Source: Diabetologia. 2026 Apr 17;69(7):2029–43. doi: 10.1007/s00125-026-06729-y (PMC13236735; doi:10.1007/s00125-026-06729-y)
Supplement: Supplementary file 1 — ESM (PDF 296 KB) [file 125_2026_6729_MOESM1_ESM.pdf]

Short-Term Modulation of Human Milk Oligosaccharides in Plasma and Milk by Glucose and Insulin: Insights into Postpartum Metabolic Regulation

Lukas Schönbacher, Anna M. Walzl, Christina Stern, Harald C. Köfeler, Harald Sourij, Herbert Fluhr, Evelyn Jantscher-Krenn, Maria A. Ramos-Roman

ESM Table 1. Concentrations of fasting plasma oligosaccharides during the OGTT in relation to GDM status.

	Total<br>*N*=28	GDM<br>*n*=16	NGT<br>*n*=12	*p*
2’FL, nmol/ml	0.77 (0.44 - 1.46)	0.77 (0.46 - 1.44)	0.72 (0.41 - 1.54)	0.98
3’SLN, nmol/ml	0.12 (0.10 - 0.13)	0.12 (0.10 - 0.13)	0.12 (0.11 - 0.14)	0.48
LDFT, nmol/ml	0.15 (0.08 - 0.29)	0.16 (0.08 - 0.29)	0.15 (0.08 - 0.39)	0.87
3’SL, nmol/ml	0.22 (0.18 - 0.26)	0.21 (0.18 - 0.26)	0.22 (0.19 - 0.29)	0.48
6’SLN, nmol/ml	0.08 (0.06 - 0.13)	0.08 (0.05 - 0.13)	0.10 (0.06 - 0.13)	0.77
LNFP1, nmol/ml	0.13 (0.07 - 0.18)	0.11 (0.05 - 0.19)	0.14 (0.11 - 0.18)	0.35
LNFP2/3, nmol/ml	0.03 (0.00 - 0.07)	0.04 (0.00 - 0.06)	0.03 (0.00 - 0.13)	0.77
LNDFH, nmol/ml	0.05 (0.02 - 0.11)	0.05 (0.01 - 0.11)	0.05 (0.03 - 0.14)	0.60

Median (IQR) and Mann-Whitney U test

ESM Table 2. Change in milk volume during the hyperinsulinaemic-euglycaemic clamp.

		p			
Breastfeeding by glucose tolerance and insulin infusion rate (IIR)	*n*	Milk 2, 3, 4	Milk 2 vs Milk 3	Milk 3 vs Milk 4	Milk 2 vs Milk 4
All (GDM plus NGT) (IIR 0/10/20 plus IIR 0/10/40)	18	0.0011**	0.2191	0.0040**	0.0090**
All (GDM plus NGT) (IIR 0/10/20)	12	0.0247*	0.7384	0.0740	0.0781
All (GDM plus NGT) (IIR 0/10/40)	6	0.0411*	0.3038	0.0727	0.0841
GDM only (IIR 0/10/20 plus IIR 0/10/40)	12	0.0027**	0.3420	0.0040**	0.0128*
GDM only (IIR 0/10/20)	9	0.0061**	0.8318	0.0687	0.0262*
GDM only (IIR 0/10/40)	3	0.1417	0.4447	0.0470*	0.2082
NGT only (IIR 0/10/20 plus IIR 0/10/40)	6	0.3941	0.6981	0.7001	0.7168
NGT only (IIR 0/10/20)	3	Unable to test, only 1 of 3 participants had the complete set of milk volumes			
NGT only (IIR 0/10/40)	3	0.3077	0.8424	0.7859	0.5419

Milk was collected on 4 separate times during the hyperinsulinaemic-euglycaemic clamp. Milk pumping was restricted to the first 20 min at the start of each stage (0-20, 120-140, 240-260 min) and from 360-370 min while still on the insulin infusion. Volume was analysed as ml/min to take into consideration that the last period of milk collection was shorter. We observed a decrease in milk volume over time that was driven by the participants with a recent history of GDM (67% of the breastfeeding participants). Milk 1 was not used in this analysis. Milk 2 was collected after 120 min in the basal period (120-140 min, representing no exposure to exogenous insulin). Milk 3 was collected after 120 min of an insulin infusion rate (IIR) of 10 mU/m^2/min (240-260 min). Milk 4 was collected after 120 min of an IIR of 20 m^2/min or 40 mU/m^2/min (360-370 min). This analysis was carried out using a mixed model. *p<0.05, **p<0.01
